# Forest point cloud registration: a review

**DOI:** 10.48130/forres-0024-0015

**Published:** 2024-05-08

**Authors:** Jincheng Liu, Yijun Guo, Juntao Yang, Ningning Zhu, Wenxia Dai, Qiang Yu

**Affiliations:** 1 College of Natural Resources and Environment, Northwest A&F University, Yangling 712100, China; 2 College of Geodesy and Geomatics, Shandong University of Science and Technology, Qingdao 266590, China; 3 State Key Laboratory of Information Engineering in Surveying, Mapping and Remote Sensing, Wuhan University, Wuhan 430079, China; 4 School of Geography and Information Engineering, China University of Geosciences, Wuhan 430074, China; 5 State Key Laboratory of Soil Erosion and Dryland Farming on the Loess Plateau, Institute of Soil and Water Conservation, Northwest A&F University, Yangling 712100, China

**Keywords:** Forestry, Point cloud, Registration, Feature matching, Multi-source platforms

## Abstract

Point cloud registration is a necessary prerequisite for conducting precise, large-scale forest surveys and management. This paper focuses on providing a systematic overview and summary of the work on forest point cloud registration over the past 20 years. The developmental process of forest point cloud registration methods, spanning from the early reliance on manual markers to the subsequent evolution towards automatic registration based on feature matching, and then to the advanced technology based on deep learning were reviewed. Furthermore, the paper offered detailed discussions on the registration between different point cloud platforms: ground platforms, between ground platforms and aerial platforms, and between aerial platforms. Additionally, the paper delved into mainstream datasets and evaluation metrics in the domain of forest point cloud registration. Finally, the paper summarized the current state of research in this area, highlighted challenges, and provided future research outlooks. This review aims to provide researchers with a comprehensive understanding of forest point cloud registration, and to promote the advancement of point cloud technology, hopefully inspiring further applications in the field.

## Introduction

Forests are recognized as the largest terrestrial ecosystems on Earth. They not only provide vital resources for the economy and human well-being, but also play a crucial role in maintaining the equilibrium and stability of other global ecological systems^[1,2]^. Hence, precise measurement and monitoring of forests are of paramount importance. Traditional methods perform well on a small scale but face limitations regarding time, spatial resolution, and efficiency when extended to larger areas.

As photogrammetry and remote sensing technologies continue to advance, the benefits of three-dimensional point cloud data in reconstructing forest scenes and extracting feature information are becoming increasingly evident. Light Detection and Ranging (LiDAR), renowned for its high accuracy and simplicity of operation, has emerged as the preferred method for acquiring point cloud data across various domains within the realm of remote sensing. However, with the advent and ongoing refinement of various image matching and point cloud reconstruction algorithms, optical imagery is steadily gaining ground as a primary source of point cloud data^[3,4]^.

In practical applications, catering to diverse forest inventory tasks often involves extracting relevant factors independently from various datasets. Moreover, substantial repetitive surveying may be required to compensate for data gaps within a single dataset. However, differences in data acquisition platforms across datasets introduce challenges such as spatial scale discrepancies, variations in density, accuracy, and detail representation, even within the same forest stand. As a result, establishing correlation among feature factors across multiple platform datasets becomes arduous^[5]^. Therefore, registering collected point cloud data is imperative to ensure consistency.

Forest point cloud registration (PCR) aims to register point cloud data collected from various perspectives, times, and platforms to construct a comprehensive three-dimensional model of the forest. Therefore, it has garnered significant attention in the forestry sector, notwithstanding encountering several technical and practical challenges. Firstly, due to the dense and irregular nature of forests, noise is often prevalent in point cloud data, particularly in image-based point clouds. Secondly, factors such as occlusion in forest environments can lead to missing data in point cloud datasets. Additionally, seasonal variations and other natural factors may introduce inconsistencies between data collected at different times, thereby complicating the registration process. Furthermore, disparities in data quality and resolution among different platforms further complicate the registration process. Moreover, the absence of unified evaluation standards hampers the development and application of registration algorithms. Lastly, PCR primarily operates at the plot scale, limiting its applicability in large-scale scenes.

In response to these challenges, this paper endeavors to present a comprehensive review of research on forest PCR over the last two decades. We categorized and summarized the various existing methods, offering insights into future research directions. Our objective is to provide valuable references for researchers and contribute to the advancement of efficient forest resource management and conservation.

## Status of research and development trends

In the field of forest ecology research and resource management, accurately acquiring and analyzing high-precision three-dimensional structural information is crucial for precisely representing the morphology, structure, and dynamic changes of forests. Through the utilization of LiDAR technologies such as Terrestrial Laser Scanning (TLS), Backpack Laser Scanning (BLS), Mobile Laser Scanning (MLS), Airborne Laser Scanning (ALS), Unmanned Aerial Vehicle Laser Scanning (ULS), along with unmanned aerial vehicles and ground-based photogrammetry, researchers can capture the three-dimensional spatial structure of forests with unparalleled detail and accuracy. PCR serves as an indispensable step in the amalgamation of these technologies. Within the forest environment, PCR has progressed through three primary stages of development: initially relying on artificial markers, transitioning to the prevalence of feature-based methods in recent years, and currently moving towards the modern trend of development based on deep learning methods.

### Methods based on artificial markers

In the early days, PCR commonly involved positioning artificial markers, such as reflector balls or artificial reflectors, within sample plots. Registration was then conducted through manual identification of these markers or by utilizing software^[6−8]^. Hilker et al.^[9]^ introduced four connections within the sample plots to improve the precision and reliability of registration. Subsequently, Pueschel^[10]^ utilized four FARO laser scanner reference spheres and one planar target for manual registration, achieving sub-millimeter accuracy. To simplify the process of using artificial markers, Zhang et al.^[11]^ applied the back sighting orientation approach, using only one reflector as a connection point between two scans. During this stage, researchers attempted to enhance the registration precision by improving the placement and detection methods of manual markers. While offering advantages such as high accuracy and strong reliability, the process of setting up markers requires considerable time and effort, and user intervention during marker identification may be necessary^[12]^, thus limiting their practicality and efficiency.

### Methods based on feature matching

These methods identify and extract key features from the forest point cloud data. Subsequently, the required transformation parameters are estimated by registering the identified features, ensuring the uniform registration of individual point clouds into a common frame of reference. Due to the irregularity of forest point clouds and the frequent self-similarity in their surrounding regions, registration methods characterized by geometric elements (points, lines, and surfaces) are ineffective in forests. In forest scenes, tree stems are typically considered the most stable structures. Hence, some researchers have chosen tree location^[13,14]^, the correlation between tree height and diameter at breast height^[15]^, stem position or stem curvature^[16−18]^, digital terrain models^[19]^, and canopy height models^[20]^ as distinctive feature points for the registration process. At the same time, researchers employed specific descriptors for a more sophisticated feature matching approach. Descriptors such as Fast Point Feature Histogram (FPFH)^[21]^ and YOHO-Desc^[22]^ capture local shape information in point cloud data, aiding in the registration process. Furthermore, researchers have explored the creation of registration primitives using various feature combinations, including tree locations and inter-tree distances^[23]^, key points and stem locations extracted from tree crowns^[24]^, or shaded regions identified in the original point cloud from a single scan^[25]^. Most contemporary forest PCR methods are divided into two separate steps, coarse and fine registration. Coarse registration is achieved through feature matching to obtain a better initial pose, while fine registration further refines the registration error using algorithms such as Iterative Closest Point (ICP). Feature-based methods can automatically detect and match feature points, making them widely applicable. However, they are sensitive to point cloud quality and environmental changes, often requiring data preprocessing, and parameter tuning for these algorithms may be cumbersome. Additionally, while feature-matching methods can handle some level of noise and local deformation, they may encounter issues such as mismatches and omissions.

### Methods based on deep learning

With breakthrough advancements across various domains in recent years, deep learning technology has gradually been introduced into forest PCR research. The PointNet and PointNet++ networks provide a robust foundation for deep feature extraction and classification of point cloud data^[26]^. Leveraging these advanced network architectures, researchers can more accurately extract key features from forest point cloud data. Wang et al.^[22]^ achieved a registration success rate of 99.8% by designing a learning-based sparse graph construction and a pose graph solving strategy based on historical reweighting. Although learning-based methods can automatically learn features and handle complex large-scale data, they require a large amount of annotated data for model training and have high computational complexity, potentially facing overfitting issues.

As PCR technology is increasingly applied in forest resource monitoring, the field of forest PCR is undergoing a transition from manual marker methods to automation and artificial intelligence. Each method has its advantages and limitations, as depicted in Table 1, and the choice should be based on specific circumstances. In complex forest environments and in research involving point cloud segmentation, classification, etc., manual marker methods remain indispensable. In relatively simple or moderately complex forest environments, feature-based matching methods have matured, but there are differences in feature point extraction and matching. Learning-based methods are still in their early stages, but their potential is promising. With ongoing technological advancements, it is anticipated that future forest PCR will become more efficient, accurate, and intelligent.

**Table 1:** Advantages and disadvantages of different PCR methods.

Method	Advantages	Disadvantages
Artificial markers	1. High accuracy	1. Placement of markers is limited, requiring uniform distribution
	2. Strong reliability	2. Setting up markers is time-consuming and labor-intensive
	3. Strong controllability	3. User intervention might be needed during marker identification
Feature matching	1. Automatically detects feature points	1. Sensitive to point cloud quality and environmental changes
	2. Broad applicability	2. Typically requires preprocessing of point cloud data
	3. Handle noise and local distortion	3. Parameters of algorithms need experimentation and adjustment
	4. Integrate with other technologies	4. May encounter mismatches and omissions
Deep learning	1. Automatically learns features	1. Requires a large amount of annotated data for model training
	2. handle complex, large-scale data	2. High computational complexity, may face overfitting issues

## Forest PCR methods based on multi-source platforms

**Figure 1:**
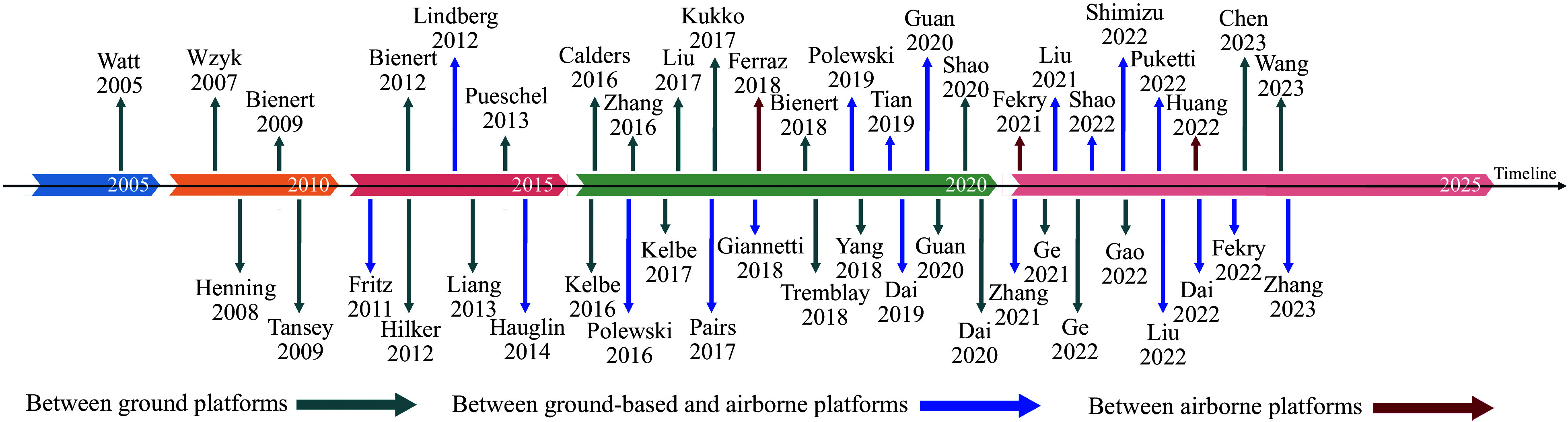
Trend of forest PCR methods based on different platform architectures.

In the practical application of forest PCR, the registration issues and tasks vary among different platforms, necessitating the adoption of diverse registration methods. With this consideration in mind, searches using keywords such as 'forest' and 'point cloud registration' in databases including CNKI, WANFANG, and Web of Science were conducted, summarizing and synthesizing relevant literature from approximately 2003 to June 2023. After reviewing the majority of relevant literature and considering practical application scenarios for forest PCR, platform architecture was categorized as follows: between ground platforms, between ground platforms and aerial platforms, or between aerial platforms. Additionally, Fig. 1 was plotted to visually illustrate the trend of forest PCR methods based on different platform architectures for readers' reference.

### Methods based on registration between ground platforms

#### Methods for registration between TLS and TLS

(1) Methods based on artificial markers

Periodic forest PCR methods typically involve the placement of artificial markers such as reflective spheres and artificial reflectors in sample plots. Subsequently, the registration process occurs either through manual identification of these markers or *via* software^[8,9,27,28]^. Bienert & Maas^[28]^ discussed two registration methods: one utilizing white spheres as connectors, and the other relying on extracted tree axes and a connector. While both methods visually seem to achieve registration, specific quantitative metrics are needed to assess their effectiveness. Hilker et al.^[9]^ placed four 0.20 m diameter polystyrene spheres in each sample plot to register multiple TLS scans, resulting in Root Mean Square Errors (RMSE) of registration ranging from 0.04 to 0.7 m. To reduce reliance on artificial markers, Zhang et al.^[11]^ employed the back sighting orientation technique between adjacent scans for coarse registration, followed by fine registration using the centers of tree stems. The results indicated a mean error of 0.18 m for coarse registration and 0.015 m for fine registration. Additionally, Giannetti et al.^[29]^ achieved registration accuracy of 0.002 m by automatically detecting spherical targets using Trimble RealWorks^[18]^ software. Despite its high accuracy, ground platform registration based on artificial markers has limited potential for improvement due to the time-consuming and labor-intensive process of marker placement and identification.

(2) Methods based on feature matching

Given that tree stems are the most stable structures in forest scenes, most automated forest PCR methods rely on extracted attributes of tree stems for registration. For example, Liang & Hyyppa^[14]^ performed registration of a forest point cloud based on stem locations by determining all possible matching vector pairs. The overall stem detection accuracy after registration was 95.3%. Although this approach is theoretically feasible, it does not provide a specific quantitative assessment of registration accuracy, and the computational complexity increases with the number of unknown parameters. Kelbe et al.^[16]^ eliminated dissimilar stem-triplet pairs using a similarity assessment of Diameter at Breast Height and geometric feature descriptors derived from the stems, resulting an average error of 0.076 m horizontally and 0.124 m vertically after registration. Building on the work of Liang & Hyyppa^[14]^, Liu et al.^[17]^ further addressed the registration problem by reconstructing the stem curves from each TLS scan and then matching the trunks at the feature level. The RMSE values for horizontal and vertical translations were 0.016 and 0.131 m. Tremblay & Beland^[18]^ expanded on the work of Kelbe et al.^[16]^ by comparing the lengths of the vertices of the stem triangles and the diameter at breast height to establish inter-stem similarity for registration, which resulted in average errors in the x, y, and z directions of 0.014, 0.013, and 0.08 m, respectively. Compared to Kelbe et al.^[16]^, the accuracy in the horizontal direction was significantly improved, and the algorithm execution time was reduced. However, as the number of stems in each scan increases, the execution time of these methods rises rapidly. Therefore, researchers explored alternative features for registration. Yang et al.^[21]^ used the FPFH approach to compute the initial transformation matrix based on a sample-consistent initial registration algorithm for coarse registration. Then, Normal Distributions Transform (NDT) was employed for fine registration. The results indicated that the FPFH-NDT algorithm has an average registration error of 0.323 m, outperforming FPFH-ICP, NDT, and ICP. Guan et al.^[25]^ used shaded regions in the original point cloud data from a single TLS scan as a key feature for registering multiple scans of TLS data. The results showed that the average residual of the registration was 0.042 m, comparable to manual registration methods. However, the computational time of the enumeration process increases exponentially as the number of identified visually occluded points increases. Dai et al.^[24]^ employed semantically guided key point detection and a robust random sampling consistency mechanism to enhance the accuracy and efficiency of registration, achieving an average residual distance of 0.049 m.

(3) Multi-view methods

When performing ground-based LiDAR PCR, several forest registration methods adopt a sequential framework^[15,25,30,31]^. However, each registration introduces a certain amount of error, and these errors accumulate with multiple registration steps. To further minimize registration errors, Ge & Zhu^[30]^ proposed an automated end-to-end framework, RegisMUF, for registering unordered forest point clouds. The procedure includes the following steps: 1) Generate a reliable scanning network by predicting overlapping regions in possible neighboring scans based on a common subgraph. 2) Apply a pairwise approach to all connected scans at a coarse level, and then verify the integrity of the frames using a pose test. 3) Refine the matching process by applying an enhanced fine registration method under an accelerated network convergence strategy. The results indicated that, for the tested dataset, the rotational error was below 0.2°, and the translational error was less than 0.2 m. Subsequently, Wang et al.^[32]^ proposed a method that combines reliable pose graph initialization and history reweighting. The specific steps are as follows: 1) Construct a sparse pose graph utilizing neural networks to learn the global features of each point cloud, estimate the overlap ratio between scan pairs by comparing the correlation of two global features, and use this information to build a sparse but reliable pose graph (containing fewer but more trustworthy edges); 2) Design an iteratively reweight a least squares scheme with a history reweighting iterative scheme: Initialize edge weights based on global features and registration results, and then iteratively refine poses using the history reweighting function; 3) Conduct point cloud registration: Apply registration algorithms on the sparse pose graph, perform registration calculations only on the edges in the sparse graph, and then use the proposed IRLS scheme to optimize and synchronize the global poses of all scans, achieving overall registration consistency and accuracy.

#### Registration among other ground platforms

The registration among ground platforms mainly occurs between TLS. However, there are also cases involving other platforms, such as TLS and BLS^[33,34]^, or BLS and BLS^[35]^, although research in this area is less common. Kukko et al.^[36]^ conducted registration with MLS, involving the following key steps: 1) Initial trajectory acquisition utilizing the Global Navigation Satellite System (GNSS) and Inertial Measurement Unit (IMU) to obtain the initial trajectory of the MLS system. 2) Feature detection and matching employing data collected by MLS to identify and match features such as tree trunk positions in the environment. 3) Application of the Graph SLAM algorithm to correct geometric inconsistencies induced by GNSS-IMU observation data. Based on tree trunk feature positioning data, the internal consistency of the data was significantly improved from 0.7 to 0.01 m. When compared with plot references, the average absolute tree stem position error was reduced to 0.06 m. Gao et al.^[35]^ applied various algorithms including ICP, NDT, curvature-based point cloud matching, PFH feature-based matching, FPFH feature-based matching, and 3DSC feature-based matching for the registration of BLS point clouds for different scenes. The findings revealed that BLS registration is notably influenced by slopes, with the ICP algorithm being the least affected. The FPFH feature-based matching algorithm performs best in areas with smaller slopes, while the PFH feature-based matching algorithm achieves the highest registration accuracy on steeper terrains.

### Methods based on registration between ground platforms and aerial platforms registration

In the early stages, the registration between ground and airborne platforms involved registering the coordinate systems of field measurements with aerial data coordinates^[13,37]^, meeting the initial requirements for forest resource monitoring. Over time, the demand for comprehensive three-dimensional structural information on forests has grown. Ground platforms can provide detailed information on the lower canopy structure but have a limited perspective on the upper canopy. By contrast, airborne platforms can capture detailed information on the top canopy but face obstacles when penetrating the lower canopy. Registering the data from both platforms helps construct a complete and detailed three-dimensional forest structure, providing a comprehensive perspective for forest management and research.

#### Methods for registration between TLS and ALS

Over the past decade, the registration of TLS and ALS has evolved from early attempts toward more advanced two-step registration^[38,39]^. In the initial endeavors, tree matching rates were used as an assessment metric for TLS-ALS registration, but this indicator only provided a preliminary evaluation of the registration quality, lacking precision. Fritz et al.^[40]^ proposed registration methods using simple geometric features and tree-to-tree distances. This study presented three methods as follows: 1) Focused on planar distance-based registration by calculating distances between trees; 2) Employed three-dimensional line fitting techniques, utilizing trunk information from TLS to estimate potential tree top positions in ALS; 3) Involved cross-registration based on three-dimensional box models. It used estimated tree top positions, geometric centers from TLS, as well as tree crown diameter and height information from ALS to create two 3D box models, determining matching possibilities by checking if they intersected. Despite their simplicity, all three methods exhibited low registration accuracy, with tree matching rates below 50%. Lindberg et al.^[41]^ built upon the tree-matching algorithm proposed by Olofsson et al.^[13]^ and directly identified trunk features in TLS for registration. While this approach improved registration to some extent, accuracy remained limited, especially when tree stems were obscured or trees were in close proximity.

Subsequently, researchers employed more advanced features or a combination of various features to enhance registration accuracy. Hauglin et al.^[15]^ utilized normalized features of DBH and tree height for registration. Paris et al.^[20]^ employed CHM as features for registration. The former exhibited lower registration accuracy, with an average of 84% of data having registration errors within 1 m. The latter showed CHM image correlation coefficients ranging from 0.53 to 0.73 after registration, with RMSE of estimated tree height and crown width reaching 0.39 and 1.46 m, respectively. However, both evaluations were conducted in the horizontal direction, with the vertical registration issues left unresolved. Additionally, these methods were only suitable for more open forest areas, requiring further improvements for dense forest registration.

In the past, the registration of TLS and ALS primarily relied on feature-matching methods, such as tree positions or shapes^[42]^. However, with increasing demand for registration and technological advancements, the registration process has evolved into a two-stage strategy. This involves initially establishing a rough correspondence through coarse registration, followed by fine registration based on this initial registration. For instance, Giannetti et al.^[29]^ employed visual inspection, treating trees as corresponding points, for rough registration using Cloud Compare software. Subsequently, Trimble Real Works software^[18]^ was utilized for a blended multi-view adjustment of the digital terrain model extracted from the point cloud. Further fine registration was conducted with ALS using the reference coordinate system. The results indicated a coarse registration accuracy of 0.05 m, while the fine registration accuracy, assessed by terrain height difference indicators, had RMSE values of 0.02 and 0.03 m for TLS, HMLS, and ALS. Although this registration method demonstrated high accuracy, its applicability was limited due to its reliance on manual markers. Given the irregular distribution of natural forest point clouds, Dai et al.^[43]^ proposed a density-based registration method. This approach utilizes a probability framework and maximum likelihood estimation for registration, involving the following main steps: 1) Minimizing the difference in point density between ALS and TLS crown points to generate similar pattern points. These pattern points represent local maxima of potential probability density functions. 2) Registering pattern-based key points using the Coherent Point Drift algorithm. 3) Applying the recovered transformation to the original point cloud and optimizing it using the standard ICP algorithm. The results demonstrated that the proposed probability-based method performed well, with a 3D distance residual of 0.07 m. Notably, this method avoided the need for descriptor similarity around key points or a single-tree segmentation process, but its effectiveness could be influenced by crown overlap.

#### Methods for registration between TLS and ULS

Several recent studies on TLS and ULS PCR promoted the evolution from registration based on simple features to the development of complex algorithms^[44−47]^. The proposed tree height registration method relies on Singular Value Decomposition (SVD) for coarse registration and achieves fine registration through iterative SVD^[44]^. The results showed an average accuracy of 0.43 m. To reduce dependence on tree attributes and improve registration efficiency, Zhang et al.^[45]^ employed FPFH for coarse registration, after which they used the ICP algorithm for fine registration, and ultimately eliminated cumulative errors in overlapping regions of multiple scan point clouds through a graph-based adjustment framework for seamless connection. The results indicated that after registration, the horizontal RMSE values ranged from 0.02 to 0.03 m, while vertical RMSE values fell within the range of 0.010 to 0.015 m. This suggests that the method performs well in terms of registration accuracy. However, the parameter selection of the FPFH algorithm significantly influences the quality of the registration results, with inappropriate parameter settings leading to unstable registration quality. To overcome this, Liu et al.^[46]^ proposed a registration method based on feature triangles, involving the following main steps: 1) Tree positions are extracted from both ULS and TLS; 2) Using tree positions as registration primitives, feature triangles are constructed, and same-name triangle pairs are identified based on similarity metrics; 3) Using these identified triangles, computed registration transformation parameters are applied. The average registration accuracy of this method is 0.31 m, demonstrating relatively high positional accuracy. However, the registration performance may decrease with an increase of forest density, which may result in significant errors in local areas. In the same year, Shao et al.^[48]^ also introduced an efficient automated registration method that primarily involves the following steps: 1) Ground registration, adjusting ground point clouds to the horizontal using ground filtering and a Random Sample Consensus algorithm; 2) Crown registration, projecting vegetation point clouds to form a 2D image and applying morphological operations; 3) Image matching, performing key point matching based on crown shape context features; 4) Point cloud registration, finely adjusting rotation and translation matrices using the ICP algorithm for registration. However, the results indicated that the average accuracy of coarse registration did not exceed 0.2 m, and after fine registration, the average distance and RMSE values were less than 0.15 m. Moreover, for samples with high canopy density, using all canopy points may not provide sufficient valid information for accurate fine registration. Additionally, the setting of the canopy projection range is based on empirical knowledge rather than an adaptive or automated approach, limiting its universality for different forest structures.

#### Methods for registration between BLS and ULS

TLS and BLS both fall under the category of ground laser scanning technologies. TLS holds an advantage in stability and accuracy, while BLS, utilizing SLAM technology, can precisely stitch together scanned point cloud data without the need for ground control points. This significantly reduces the workload and time required for setting up ground control points in the field, simplifying the operational process and decreasing reliance on heavy equipment, making data collection in challenging or sensitive forest environments more feasible and convenient. Polewski et al.^[49]^ employed tree-to-tree distances as features to register BLS and ULS point clouds. The method involves the following key steps: 1) Adjusting the Z-axis of the point cloud by leveraging the negative gravitropism of tree growth; 2) Construction of relative features: Using plane distance metrics to construct descriptor vectors between trees; 3) Graph matching and similarity assessment: Establishing a bipartite graph and employing the Kuhn-Munkres algorithm to find the best matches; 4) Scale estimation: Maximizing the graph matching score function to estimate the relative scale between datasets; 5) Optimal transformation search: Utilizing simulated annealing to find the matching subset leading to the best coordinate transformation; 6) Scale refinement: Fine-tuning the scale factor through a detailed grid search and selecting the optimal result.

#### Methods for registering ground-based and ULS with ground-based and UAV image point clouds

Combining the method of laser PCR with the integration of image point clouds significantly reduces the cost of forest measurement^[23,50,51]^. On the operational front, it simplifies the data collection process, enabling even non-professionals to collect and process data with relative ease. Additionally, it offers advantages in improving spatial resolution and data update frequencies, greatly enhancing the timeliness and credibility of forest resource monitoring data. It provides a more efficient and reliable technical approach for the accurate assessment and management of forest resources. Polewski et al.^[23]^ introduced a method for registering ground photogrammetric point clouds with ALS in forested areas. This method uses tree positions as registration primitives, measuring similarity by calculating horizontal and vertical distances between each tree and its surrounding trees. The main steps include: 1) Estimating tree stem positions in-ground and ALS point clouds, constructing descriptors to represent the plane and vertical distances to other trees; 2) Computing descriptors: Calculating the similarity of all stem descriptor pairs between the two point clouds and determining corresponding trunk pairs using graph matching techniques; 3) Finding the optimal transformation: Selecting the best set of trunk pairs from the matched dataset using a heuristic algorithm; 4) Constructing stem descriptors: Characterizing the distance of each stem relative to other stems within the same plot; 5) Quantifying stem descriptors: Applying a radial basis function to measure the similarity between descriptors; 6) Graph matching: Finding the best matches for trunks based on descriptor similarity; 7) Determining rigid transformation: Computing transformation parameters for optimal registration of the two datasets; 8) Identifying associated point subset: Selecting the optimal subset from the results of graph matching for coordinate transformation. The results demonstrated that this method achieved an average registration accuracy of 0.66 m, confirming the feasibility and effectiveness of applying ground photogrammetric point clouds in forest scenes. At the same time, the high spatiotemporal resolution and strong maneuverability of UAVs have made them an effective complement in the field of aerial photogrammetry. Tian et al.^[50]^ explored the possibility of using low-cost UAVs to replace ALS for forest measurements. This method, relying on distinct landmark contour features and ground control points, achieved a registration accuracy of 0.06 m through precise geometric correction, significantly enhancing the application potential of UAVs in forest mapping.

### Methods for registration between aerial platforms

In general, data registration between aerial platforms has not been widely explored in forest PCR. Nevertheless, utilizing aerial platforms for long time-series data holds crucial value for monitoring the growth changes in forests and conducting refined resource management. Due to significant differences among aerial platforms in flight altitude, scanning angle, coverage area, and data density, the registration process faces notable challenges. Ferraz et al.^[52]^ achieved the registration of ALS point clouds from different time points by taking advantage of the stable features of coniferous tree tips for time-variant analysis. The results indicate that this method effectively reduces estimation biases to 0.38 m in horizontal and 0.12 m in vertical direction, mitigating spatial discrepancies caused by platform trajectory and motion uncertainties. Fekry et al.^[53]^ proposed a comprehensive strategy for forested areas using automated UAV-mounted LiDAR with adjustment based on hierarchical density clustering analysis of vegetation cover. The method consists of three key stages: 1) The scanned vegetation cover is clustered using the HDBSCAN algorithm, and key points are labeled by employing topological persistence analysis for each cluster; 2) Feature similarity is computed by taking into account the linear and angular relationships between each point and the centroid of the point set. The Kuhn-Munkres algorithm is then utilized to address the assignment problem based on the similarity score function, resulting in a set of matched pairs that establish one-to-one feature correspondences. 3) The 3D rigid transformation parameters are determined by exploring all potential one-to-one pairings within the correspondence. The optimal pairing is defined as the set that includes the maximum number of matching points, all adhering to a specified tolerance for distance residuals. Due to their cost-effectiveness, point clouds generated using UAV photogrammetry are increasingly common in related studies. Huang et al. employed the Global Registration Method Using a Robust 61 Phase Correlation method for registering TLS and UAV image point clouds^[54]^. The main steps include: 1) Voxelizing point clouds into three-dimensional cubic signals and transforming them into the frequency domain to estimate phase differences. 2) Calculating the linear function coefficients between phase difference angles and displacement parameters, followed by the application of a robust estimator to solve linear equations. As a result, the method effectively estimates three-dimensional offsets. By leveraging the low-frequency components of the normalized cross-power spectrum, this approach efficiently filters out noise and unrelated parts from the signal, making it adaptable for PCR tasks with low overlap and poor detail correspondence.

### Comparison and summary

Registration between ground platforms represents the most fundamental type of forest PCR, involving point clouds from the same ground scanning device at different locations or times. Challenges in this registration task include issues arising from occlusion, scanning range limitations, and uneven ground surfaces. As ground scanning provides detailed information about tree stems and the ground layer, registration algorithms typically need to identify stable reference features, such as the positions and structural attributes of specific trees.

Registration between ground and aerial platforms poses a more complex problem. Aerial platforms often offer canopy information from a top-down perspective, with less capture of details regarding tree trunks and the ground. Therefore, registering ALS and TLS data requires consideration of the geometric and scale differences between these two types of data. Researchers commonly rely on advanced feature matching and sophisticated algorithms to bridge the gaps in perspective and scale.

Registration between aerial platforms usually involves data from different flight missions or various UAV platforms. It is primarily employed for the registration of long time-series data to monitor forest growth changes and enable refined resource management. However, research on registration between aerial platforms is currently limited.

## Forest PCR benchmark datasets and evaluation criteria

### Forest PCR benchmark datasets

Forest PCR is mostly based on closed datasets curated by various research institutions and data providers. These datasets are typically sourced from forest point cloud data obtained through different platforms. To facilitate a reliable assessment and comparison of forest PCR methods, there is a pressing need for open benchmark datasets featuring a substantial data volume and diversity. Unfortunately, the currently available datasets are limited in size.

### Evaluation criteria

To impartially and effectively assess the performance of forest PCR methods, a unified evaluation framework must be established, incorporating both quantitative and qualitative analyses.

#### Qualitative metrics

Qualitative metrics play a crucial role in assessing the performance of forest PCR methods by providing intuitive detailed close-up diagrams of the registration outcomes^[25,31,43]^. In practical applications, comparing cross-sectional plots, as well as the smoothness of curves before and after registration, effectively demonstrates the continuity and consistency achieved through registration.

#### Quantitative metrics

Quantitative metrics are pivotal for evaluating the performance of forest PCR methods. These metrics can be categorized into three main types: feature extraction and matching, rigid transform estimation, and runtime. Feature extraction-based metrics include Feature-Match Recall (FMR) and Registration Recall (RR)^[32]^. Metrics based on rigid transform estimation typically include Mean Absolute Error (MAE)^[36]^, Mean Square Error (MSE), and Root Mean Square Error (RMSE)^[31,48,55]^, focusing on the rotation matrix and translation vectors in the rigid transform. These metrics evaluate the precision and accuracy of estimating the rigid transform post-registration. Finally, runtime reflects the computational efficiency and speed of the algorithm^[35,43]^.

(1) FMR

The FMR is the ratio of correct point pairs extracted from features to all the point pair data in the point cloud.

(2) RR

The RR is calculated by assessing how well a registration method recovers point clouds with overlapping regions from two sets of point clouds, both having rigid transformations and overlapping parts.

(3) MAE

The MAE is the average of the absolute errors between the true and predicted values in the rigid transformation. It can be expressed using formula (1).

1 $ MAE=\dfrac{1}{n}\sum _{i=1}^{n}\left|{y}_{i}-\overline{{y}_{i}}\right| $

where $ \overline{{y}_{i}} $ is the predicted value of the registration method, and $ {y}_{i} $ is the true value of the rigid transformation.

(4) MSE

The MSE is the average of the squared differences between the true and predicted values in the rigid transformation. It can be expressed using formula (2).

2 $ MS E=\dfrac{1}{n}\sum _{i=1}^{n}{\left({y}_{i}-\overline{{y}_{i}}\right)}^{2} $

where $ \overline{{y}_{i}} $ is the predicted value of the registration method, and $ {y}_{i} $ is the true value of the rigid transformation.

(5) RMSE

The RMSE is the average square root error between the true and predicted values in the rigid transformation. It can be expressed using formula (3).

3 $ RMS E=\sqrt{\dfrac{1}{n}\sum _{i=1}^{n}{\left({y}_{i}-\overline{{y}_{i}}\right)}^{2}} $

where $ \overline{{y}_{i}} $ is the predicted value of the registration method, and $ {y}_{i} $ is the true value of the rigid transformation.

## Future outlook

**Figure 2:**
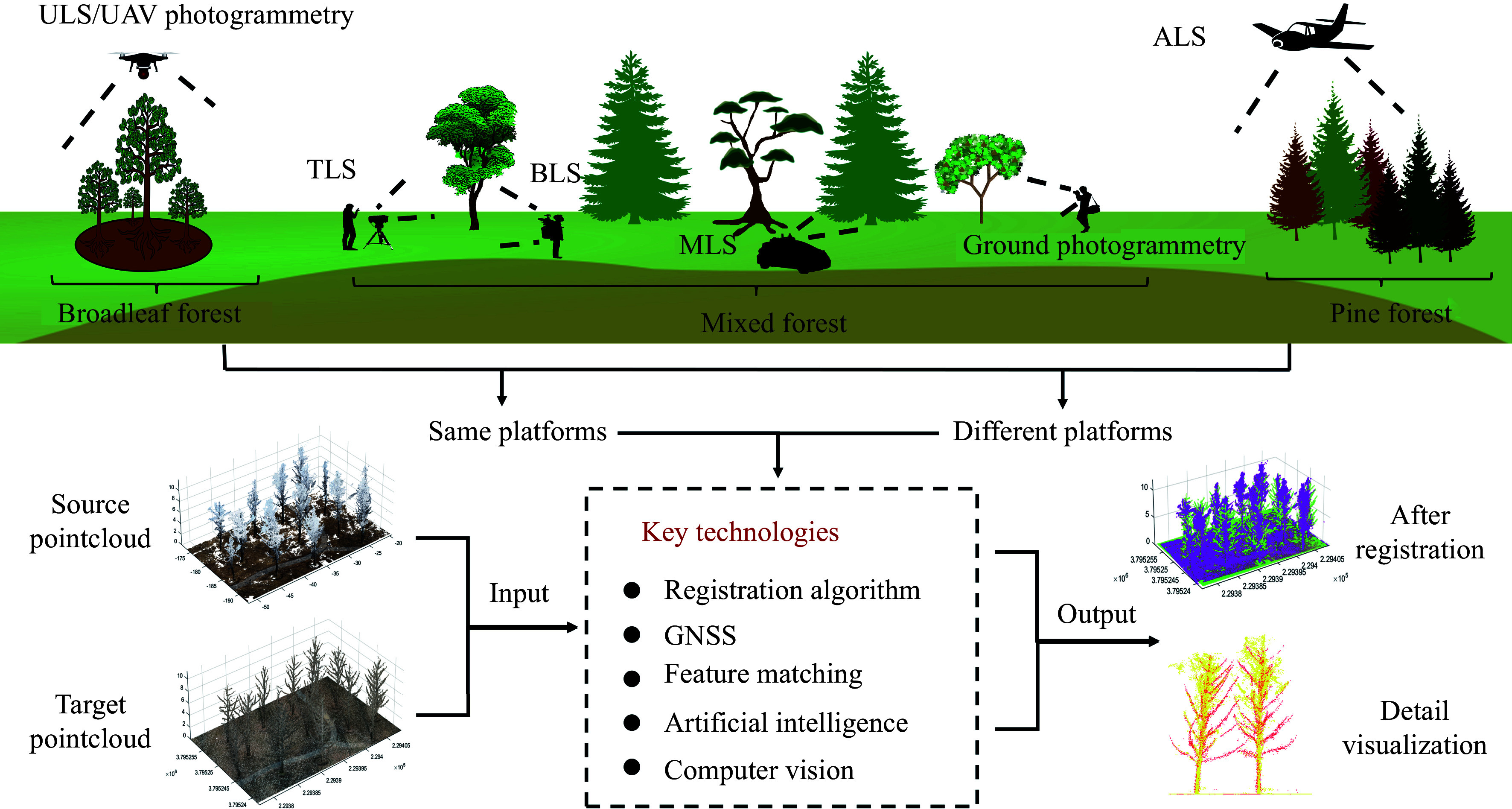
Future outlook of forest PCR.

Through an analysis of forest PCR methods across various platforms, we have obtained initial insights into the methodological framework of forest PCR. While existing methods for forest PCR are relatively mature within individual platforms, there is presently a deficiency of universal algorithms suitable for all platforms. With the ongoing advancement of registration algorithms, GNSS technology, feature matching, artificial intelligence, and computer vision, we are confident in the future advancement of forest PCR (Fig. 2). Specifically, we anticipate advancements in the following areas:

The development of high-precision, rapid, and automated forest PCR algorithms remains a critical focus of current research. Presently, forest PCR methods typically operate on small scales, employing feature matching to achieve coarse and fine registration^[16,32,43]^. However, existing methods primarily perform registration on preprocessed point clouds, which struggle to handle datasets with significant real-world noise. Additionally, point cloud downsampling is often necessary to enhance efficiency, but this may compromise registration accuracy.

The utilization of cutting-edge positioning technologies, such as GNSS and Real-Time Kinematic (RTK) positioning, represents a significant advancement^[14,56]^. Despite the persistent challenge of canopy occlusion in intricate forest environments, continuous technological progress is anticipated to enhance the signal quality and coverage of GNSS systems, thereby bolstering their resilience against interference. Consequently, we foresee these technologies evolving to become increasingly reliable and precise in forest settings and their associated applications.

The utilization of more efficient feature matching techniques is paramount. Presently, forest PCR algorithms predominantly prioritize feature point selection^[24,31,32]^. However, a universally applicable set of feature points for all platforms remains elusive. As feature-matching technology continues to evolve, we anticipate the emergence of more adaptable feature point selection methods capable of accommodating forest point cloud data from diverse platforms and complex environments.

The utilization of more efficient deep learning methods is an important trend in the future. Despite the current early stage of application of deep learning in forest PCR^[22]^, the continuous development of technology will enhance the precision, automation, and adaptability of registration. Future research directions may include constructing larger-scale forest PCR datasets to facilitate effective network training and addressing adaptability issues across various forest environments.

In addition, with the continuous advancement of computer vision, photogrammetric point clouds hold vast potential for future applications^[57,58]^. Presently, forest PCR methods are primarily focused on processing LiDAR point clouds, with limited research on photogrammetric point clouds. However, as image matching and point cloud reconstruction algorithms evolve, the advantages of low cost and high information output of image-based photogrammetric techniques will offer new possibilities for forest PCR.

## Conclusions

To help researchers face the current challenges of forest PCR, this paper first systematically classified methods developed over the past 20 years, summarized and categorized them based on the classification, introduced datasets and evaluation metrics in this field, and finally concluded the research work as follows:

This paper comprehensively classified and summarized forest PCR methods. 1) In the early stage of research, researchers used methods based on artificial markers for forest PCR. These methods have advantages such as high accuracy and reliability, but the process of placing markers is time-consuming and labor-intensive, limiting their practicality and efficiency. 2) Subsequently, researchers proposed methods based on feature matching for PCR, which has received considerable attention in the past decade. These methods can automatically detect and match feature points, with a wide range of applications. However, different methods are sensitive to point cloud quality and environmental changes, often requiring parameter tuning and data preprocessing. 3) In recent years, learning-based forest PCR algorithms have been proposed successively, mainly utilizing their powerful feature-learning capabilities as well as end-to-end training frameworks. Although these methods are still in their early stages, their potential is promising.

This paper further subdivided forest PCR methods based on the different sources of point clouds to more comprehensively explore registration issues between different platforms. 1) Registration between ground platforms is the most fundamental type of forest PCR, typically using point cloud data obtained from the same type of ground scanning equipment, mainly addressing consistency and matching between data collected at different locations or times. 2) Registration between ground and aerial platforms is a more complex task, with researchers relying on advanced feature matching and complex algorithms to bridge differences in perspective and scale. 3) Registration between aerial platforms typically involves data from different flight missions or different UAV platforms, mainly addressing inconsistencies caused by differences in flight height, scanning angle, coverage area, and data density. However, research in this area is relatively scarce.

It should be noted that we also summarized the mainstream databases and evaluation metrics in the field of forest PCR. Currently, most forest PCR algorithms lack a unified dataset and evaluation system, making it difficult to make fair comparisons among existing algorithms. The summary of databases and evaluation metrics offered in this paper can guide subsequent evaluations and comparisons of the performance of existing algorithms.

## Author contributions

The authors confirm contribution to the paper as follows: study conception and design: Liu J; data collection: Guo Y, Zhu N, Dai W; analysis and interpretation of results: Liu J, Guo Y, Yang J; draft manuscript preparation: Liu J, Guo Y, Yu Q. All authors reviewed the results and approved the final version of the manuscript.

## Data availability

All data generated or analyzed during this study are included in this published article.
